# Periapical radiographs vs cone beam CT imaging for the evaluation of peri-implant bone defects: an ex vivo study

**DOI:** 10.4317/medoral.26777

**Published:** 2025-03-23

**Authors:** Nikolaos G Tzortzakis, Spyros Damaskos, Konstantina Dimakopoulou, Emmanouil Chatzipetros, Christos Angelopoulos

**Affiliations:** 1 DDS, BSc, MSc, MSc, Periodontologist-Implantologist, Athens, Greece; 2 DDS, PhD, Assistant Professor, Department of Oral Diagnosis and Radiology, Faculty of Dentistry, National and Kapodistrian University of Athens, Athens, Greece; 3 PhD, Department of Hygiene, Epidemiology and Medical Statistics, Medical School, National and Kapodistrian University of Athens, Athens, Greece; 4 DDS, MSc, PhD, Assistant Professor, Department of Oral Diagnosis and Radiology, Faculty of Dentistry, National and Kapodistrian University of Athens, Athens, Greece; 5 DDS, MSc, PhD, Professor, Department of Oral Diagnosis and Radiology, Faculty of Dentistry, National and Kapodistrian University of Athens, Athens, Greece

## Abstract

**Background:**

Data on the radiographic interpretation of peri-implantitis is still controversial. Thus, our study aimed to: a) investigate the detectability rate of ex-vivo induced peri-implant bone defects (PBDs) between observers using two different imaging methods; Cone Beam Computed Tomography (CBCT) and Periapical Radiographs (PAs), b) investigate the observers' agreement on their ability to detect PBDs according to their level of expertise and, c) determine the sensitivity and specificity of the imaging methods used to detect induced PBDs.

**Material and Methods:**

Two dried human mandibles were used in which ten dental implants were placed and eight PBDs were created simulating clinical conditions. Radiographic examination using PAs and two CBCT modes [CBCT/N (normal/0.3mm3), and CBCT/HR (HiRes/0.15mm3)] was performed at all experimental stages. All PBDs were recorded for their dimensions using a dental periodontal probe as they were used as a gold standard (GS). Finally, 145 images (49 PAs, 48 CBCT/N, and 48 CBCT/HR) were created and evaluated by nine independent observers. Three oral radiologists (OR), three implantologists (IS), and three general practitioners (GP).

**Results:**

PBDs were detected at a higher rate by ORs compared to ISs, and GPs. However, the rate of their agreement, did not reach the nominal level of significance (z-test *p-value*> 0.05), and also between observers of the same expertise, and between the different imaging methods used: CBCT and PAs (z-test *p-value*> 0.05). In total, the sensitivity of the CBCTs and PAs method was 95% and 80.5%, respectively. While the specificity for all methods was lower, 57%, 62.2% and 50.4% for CBCT/N, CBCT/H and PAs methods, respectively.

**Conclusions:**

Although CBCT performs better than PAs in ex-vivo induced PBDs, further research is needed to evaluate if the present results can be extrapolated to other clinical scenarios and defect conFigurations.

** Key words:**Cone beam computed tomography, periapical radiograph, peri implant defect, dental implants, peri-implantitis/diagnosis, diagnostic accuracy.

## Introduction

Loss of the alveolar bone around dental implants is a prognostic indicator of peri-implantitis (1). However, this cannot be the only criterion for the prognosis and progression of the disease (2-5). Thus, dentists focused on early diagnosis and elimination or reduction of bone loss both in the early stages of osseointegration of implants and in their subsequent presence in the oral cavity (3,4,6). For this reason, 2017's World Periodontology Workshop outlined the clinical and radiographic criteria for the diagnosis and management of these conditions (7). Beyond the crucial role of clinical evaluation of peri-implantitis, the imaging method routinely used to determine and monitor peri-implant bone levels is periapical radiography (PA) (7). But studies have shown that PAs underestimate bone presence around teeth and/or implants (8-10).

Nowadays, the diagnostic practice, also implements the use of Cone Beam Computed Tomography (CBCT) in implantology as a very important and useful tool, which provides a 3D radiographic display of the irradiated volume of interest (11) resulting in an irreplaceable information for the preoperative planning of both the placement of implants (12) and for the therapeutic approach/treatment of any unpredicTable postoperative complications as well as for peri-implantitis (13). However, studies have shown that radiographic examination/evaluation of implants using CBCT can underestimate peri-implant bone loss as it is influenced by factors such as: image analysis, radiographic noise, artifacts, and technical errors or even the presence of soft tissues (14-16). Although, it has been shown that CBCT is superior to PA as it more accurately depicts the detection, classification, and measurement of peri-implant bone defects, (13) other studies support that both CBCT and PA have similar diagnostic ability and clinical value, but both are clearly influenced by the morphology of bone damage and image quality (17,18). Interestingly, it was recently shown that PAs perform better than CBCT in detecting peri-implant bone defects, especially for inter-observer agreements, particularly for experienced observers as they were more consistent in assessment than inexperienced ones (19,20).

In the light of the aforementioned studies we aim to: a) investigate the detectability rate of ex-vivo induced peri-implant bone defects (PBDs) between observers using two different imaging methods; CBCT [voxel size: 0.3mm3/normal mode (CBCT/N); 0.125mm3/HiRes mode (CBCT/HR)] and PA, b) investigate the observers' agreement on their ability to detect the aforementioned defects according to their level of expertise on the CBCT and PA images, c) determine the sensitivity and specificity of the imaging methods used to detect induced peri-implant bone defects (relative to the experience level of the observers).

## Material and Methods

Our study was approved by our institutional ethics and research committee, Department of Dentistry, School of Health Sciences, (Ref. 646/16.05.2024) being also in accordance with the Declaration of Helsinki.

For this multitasking study, two dried human mandibles were used in which dental implants were placed and PBDs were created peripherally using diamond burs. The study was carried out with the following procedures:

- Implants placement - Creation of the PBDs

**Figure 1:**
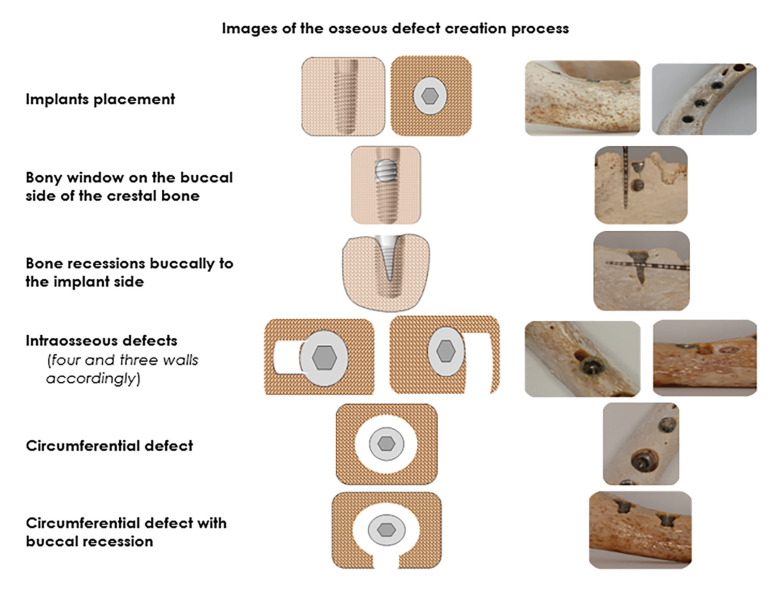
Schematic and clinical illustration of the osseous defect creation process.

Six implants PALTOP® advance (Keystone Dental Group, 154 Middlesex Turnpike, Burlington, MA 01803 USA) 3.75mm in diameter were placed in a crestal position in one mandible, and four in the other. The inter-implants distance at the implant shoulder was 4-5mm. Eight PBDs were then created, mimicking the classification of Schwarz *et al* (21) and also those encountered in clinical situations (Fig. 1): a) 1mm, and 2mm bone recessions buccally to the implant side, b) Three, and four-wall intraosseous defects, c) 1mm, and 2mm wide bony window on the buccal side of the crestal bone correspond to the implant, d) a circumferential defect around implant, and e) a similar circumferential defect with buccal recession. Note that more than one defect was created for each implant side. Moreover, the circumferential defects were created by removing the placed implant and enlarging the implantation side by using a 4.2mm wide drill (PALTOP®), leaving at least 2mm of bone intact at the apex to maintain primary stability. Similarly, the three and four-wall intraosseous defects were created using 1 and 2mm, in diameter, round burs in the crestal bone adjacent to the implants respectively, while recessions were formed using 0.5-1mm diameter conical burs. Also, prior to the X-ray examination, all defects were measured and recorded for their position and width using a dental periodontal probe (UNC-15; Hu-Friedy Inc. Leimen, Germany) to their correct dimensions according to the methodology of Garcia-Garcia M. *et al* (8). These measurements were used as the gold standard (GS). The entire procedure was performed by one of the investigators (NT).

- X-ray examination - Image processing

**Figure 2:**
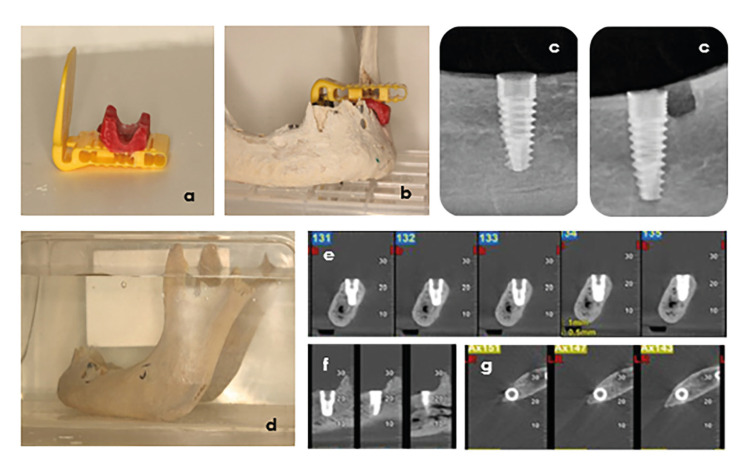
Acrylic matrix used (a,b) to maintain repeatable and stable conditions when taking periapical radiographs (c). The mandible was placed in a plastic container filled with water (d) prior to CBCT examination to imitate the X-ray attenuation produced by soft tissues. Example of CBCT images obtained in cross-sectional (e), sagittal (f), and axial (g) planes.

After placement of the implants and before the creation of PBDs, an initial radiographic examination was performed with both imaging methods (PA and CBCT). For the PA examination, a VistaIntra dental X-ray unit (Dürr Dental SE, HöpFigheimer Str. 17, 74321 Bietigheim-Bissingen, Germany) operated at 70 KVp, 7mA, (exposure time 0.12sec, focal spot size 0.4mm) and No2 phosphor plates were used. For reasons not only of reproducibility but also of X-ray beam attenuation, a radiographic acrylic splint was made that also allows the use of a parallel technic (Fig. 2). PAs were processed using the software DBSWIN 5.15 (Dürr Dental SE) (Fig. 2).

The CBCT examination was performed using a NewTom® VGi imaging unit (QR, Cefla, Verona,Italy - serial No VG17004S) at the Department of Oral Diagnosis and Radiology, School of Dentistry, NKUA. The focal spot was 0.3mm and kVp was fixed and preset at 110 kV. The mA used was variable as it was automatically determined by the machine using SafeBeam technology which allows optimal use of mA based on the density of the irradiated volume. Two imaging protocols were used: a) voxel size 0.3mm3/normal mode (CBCT/N), and b) voxel size 0.125mm3/HiRes mode (CBCT/HR). Before exposure, each dry mandible was placed in a plastic container filled with water to imitate the X-ray attenuation produced by soft tissues while reducing the contrast produced by air (22) (Fig. 2). All CBCT scans were viewed using the NNT software® (version 7.2; installation package: 7.2.0). All implants were imaged in cross-sectional (1 mm slice thickness), sagittal and axial projections (Fig. 2) while brightness, contrast, and sharpness were optimized for each image individually, for better further assessment.

- Assessment / Image comparison

Finally, 145 images (49 PAs, 48 CBCTs/N, and 48 CBCTs/HR) were created and evaluated/assessed by nine “blinded” and experienced observers (with more than three years of expertise) independently. Three oral radiologists (OR), three implantologists (IS), and three general practitioners (GP).

**Figure 3:**
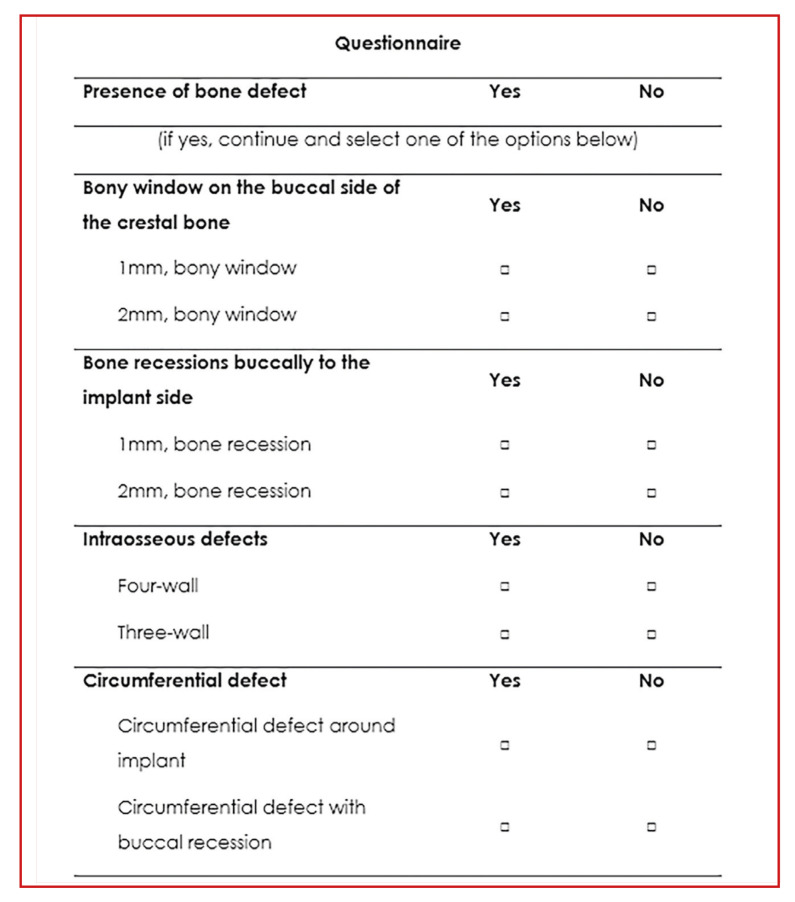
Questionnaire template.

All images were presented - using a workbook (Microsoft Excel) - in a random order and displayed on a Full-HD, 13.3” laptop screen (HP, graphic card NVIDIA, Ge Force 1050 4GB) in a PDF format to preserve their originality. The observers' response regarding the detectability of peri-implant bone defects was recorded anonymously on a specific questionnaire (Fig. 3).

- Statistical analysis

The results of the questionnaires were evaluated against the GS measurements mentioned above. The descriptive factors of the variables were examined and analyzed. Frequencies and relative frequencies were used to describe defects, both overall and for each radiographic modality separately, as well as per the level of expertise of the observers (OR, IS and GP). The Fleiss' Kappa/inter-examiner agreement assessment was applied to investigate the rate of agreement of the identification of the PBDs by different examiners with different expertise, both in the whole sample and within the same radiographic method. Then, the z-test was performed to compare the rate of agreement between the level of expertise of the observers. The same control was used to compare the rate of agreement between observers of the same expertise (e.g. IS), and between the imaging methods used [CBCT/N, CBCT/HR and PA]. Similarly, the rate of agreement of the determination of the induced PBDs with the GS was investigated according to the different expertise of the examiners in the radiographic methods used (CBCT/N, CBCT/HR and PA). Then, the sensitivity (%) and specificity (%) of the CBCT/N, CBCT/HR and PA imaging methods were calculated.

Reported *p-value*s are based on two-sided tests: *p-value*s less than 0.05 (*p*<0.05) were considered statistically significant while *p-value*s less than 0.10 were indicative (*p*<0.10). SPSSv 28 software (IBM Corp. Released 2021. IBM SPSS Statistics for Windows, Version 28.0. Armonk, NY: IBM Corp) was used for statistical analysis.

## Results

In total, 145 images (PAs and CBCTs) were created. The distribution of characteristics of PBDs submitted for evaluation, for each radiographic method (CBCT/N, CBCT/HR and PA) separately, compared to the GS, is presented in Table 1. Data shows that the percentage of PBDs detected by the CBCT imaging method (CBCT/N and CBCT/HR) is higher compared to the PA.

**Figure 4:**
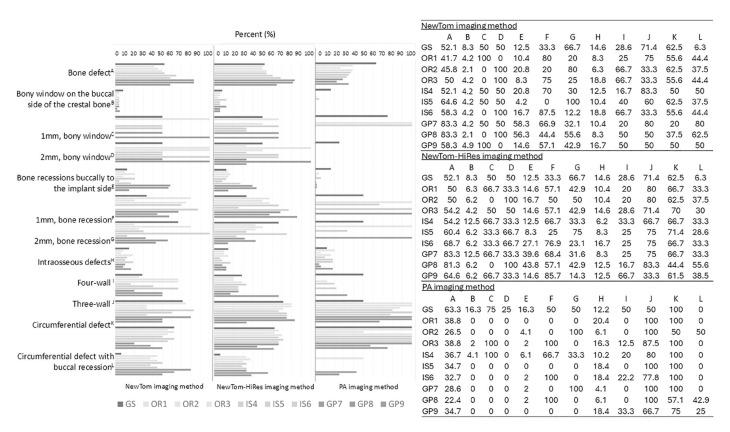
The overall detectability rate - for CBCT/N (normal mode) imaging method - of the peri-implant bone defects between observers and their level of expertise (a). The overall detectability rate - for CBCT/HR (HiRes mode) imaging method - of the peri-implant bone defects between observers and their level of expertise (b). The overall detectability rate - for PA imaging method - of the peri-implant bone defects between observers and their level of expertise (c).

The overall detectability rate (for all imaging methods used) of the PBDs between observers and their level of expertise (OR, IS, GP), is presented in Table 2. Notably, the PBDs are detected at a higher rate by ORs compared to the two other expertise’s, followed by ISs, while the lowest detectability rate was achieved by GPs. The detectability rate of PBDs between observers and their level of expertise and according to different imaging methods was also assessed (Fig. 4).

On average the percent agreement between different observers and the GS, and within the same radiographic method was higher in ORs compared to the two other expertise’s (Table 3). However, when assessing the rate of agreement, by comparing the level of expertise in pairs, the differences did not reach the nominal level of significance (Table 3; z-test *p-value*> 0.05). The same applies to the comparison of the agreement rate between observers of the same expertise, and between the different imaging methods used: CBCT and PA (Table 3; z-test *p-value*> 0.05).

The sensitivity (%) and specificity (%) of the imaging methods evaluated (CBCTs and PA) are summarized in Table 4. In total, the sensitivity of the CBCT/N, CBCT/HR and PA method was 95%, 95% and 80.5%, respectively. While the specificity for all methods was lower, 57%, 62.2% and 50.4% for CBCT/N, CBCT/HR and PA methods, respectively. In more detail, the sensitivity in all imaging methods was higher in detecting bone defects, bone recessions buccally to the implant side, intraosseous defects and cirumferential defects. Whereas the specificity of PA was higher only in the detection of a bony window on the buccal side of the crestal bone. In general, the CBCT/HR imaging method displayed the highest sensitivity and specificity in detecting defections.

## Discussion

Data on the radiographic interpretation of peri-implantitis is still controversial. Thus, our study aimed to shed light on this “hot point” of dental imaging. For this purpose, nine experienced observers of deferent level of expertise (ORs, ISs, and GPs) volunteered to evaluate images of ex-vivo created PBDs derived from different imaging methods (CBCT/N, CBCT/HR and PA).

- Evaluation of observers’ performance

Our results show that at least one of the observers, regardless of their specialty, has detected at least one defect (Table 1). In addition, our results also show that CBCT imaging methods used in our study were more susceptible to interpretation - in terms of the inability to determine the presence or absence of a bone defect - than those of PAs (Table 2). This seems to agree with the results of Song D *et al* (13) who found CBCT to be more reliable and accurate compared to PA images. However, in their study only two GPs were selected as observers, who were trained to evaluate the location (mesial, distal, buccal, and lingual) and the shape of the defect (dehiscence, horizontal, vertical, and crater-like). On the other hand, the observers in our study were of different expertise (ORs, ISs, GPs) and the experimental setting was more clinically oriented (eight defects’ shapes and 145 CBCT and PA images were evaluated). Further, investigating the effect of observers' level of expertise on defect interpretation, we found that ORs detected bone defects at a higher rate than ISs and GPs in decreasing order. Overall, inter-examiner agreement (%) supports this finding, as ORs showed almost excellent agreement, while ISs marginally excellent and GPs satisfactory agreement (Table 3). Although our methodology differs from that of Zhang CN *et al* (19), our findings also support the existing small difference in inter-observer agreements between experienced and inexperienced observers, which the authors claim.

It is worth noting that the inter-observer agreement for the CBCT/HR imaging method is almost excellent between observers, particularly ORs and ISs. The same applies - but in reduced percent agreement - to the CBCT/N. This can easily be attributed to the different voxel size (0.125mm3 vs 0.3mm3) used in the particular CBCT images. Since it has been shown that the lower the voxel size, the higher the sensitivity of the CBCT image. Note that, the latter means that the radiation dose increases significantly (20,23,24).

In the same vein, PAs evaluation showed an almost similar - very satisfactory - inter-observer agreement. This can be attributed to the observers' familiarity with this imaging method (20). Furthermore, it is of particular interest that the detectability rate of three-wall and circumferential defects is higher for PAs than any other imaging method. The same applies, reduced, for 1 and 2mm bone recession (Fig. 4).

It is worth noting that due to the inherent limitations of PA, mainly in the projection geometry (2D), these defects have a similar imaging as they are crestaly located. Our findings are in line with those of Garcia-Garcia M *et al* (8) regarding the detectability of these defects - although underestimated compared to clinical data - particularly in their mesiodistal orientation (9). In any case, the performance of PAs is directly influenced by defect size and type (18,20,25).

However, this limitation does not affect CBCT due to the 3D imaging provided. Hence, the PBDs detectability rate increases for these two methods, particularly bone recessions buccally to the implant side, bony window on the buccal side of the crestal bone and intraosseous defects. A closer look at Fig. 4. reveals that bony windows - particularly the 2mm ones - are interpreted more often in CBCT/HR than in CBCT/N. Considering that the bony windows were created using 1mm and 2mm burrs, their size affects their detectability relative to the voxel size used. The latter has been shown to produce significant results in the detectability of peri-implant defects (26,27).

To better simulate clinical settings, no observers' calibration was used in our study. However, the comparison of the rate of agreement between observers of the same, and observers of a different level of expertise did not reach the nominal level of significance (Table 3). The same applies to the comparison of the agreement rate between observers of the same expertise, and between the different imaging methods used: CBCT and PA (Table 3). All the above ensures a level of homogeneity between observers that is reflected in the accuracy of the interpretation outcomes - in terms of absence of large deviations between them - and between the different imaging methods used (CBCT and PA). Thus, we can be confident that the calculated sensitivity and the specificity of the imaging methods used are adequate as they are based on a nearly homogeneous sample of observations.

- Sensitivity and specificity

As shown in Table 4, the sensitivity of the CBCTs and PA methods was relatively high (95% and 80.5%, respectively), while the specificity for all methods was lower (57%, 62.2% and 50.4% for CBCT/N, CBCT/HR and PA methods respectively). As regards sensitivity (i.e. few false negative results), our findings are in line with those suggested by other studies who claimed that CBCT has a significantly higher diagnostic accuracy compared to PA for the detection of bony windows, recessions and three-walled intraosseous defects (13,18,20). It is worth noting that also in our study the sensitivity of all imaging methods was higher in the detection of bone defects, bone recessions buccally to the implant side, intraosseous defects and cirumferential defects. Considering that the PBDs in our study were created using round burrs of 1-2mm while recessions were formed using 0.5-1mm conical burs, these defects were wider than those of 0.675mm that Dave M *et al* claim is the threshold of a significant difference in diagnostic detection accuracy between the three imaging methods used (20). Thus, our results coincide. The same applies for the circumferential ones, as a 4.2mm wide drill was used to create a 0.45mm wide defect around the implant (4.2mm-3.75mm=0.45mm), hence this size of circumferential defect is smaller than the aforementioned threshold of 0.675mm. Reasonably, this - among others - is also attributed to the different image resolution of each imaging method used in our study [theoretical resolution: 40 l/mm and 1-2 l/mm-1 for PA and CBCT respectively] (28). As expected, PAs showed low sensitivity in detecting buccally sided bony window compared to CBCT imaging. This can easily be attributed to the inherent 2D limitations of this method. However, factors such as cortical bone thickness, exposure parameters and defect size are likely to overstress existing limitations (7,8,9).

On the other hand, specificity (i.e. few real negative results) of PA was higher only in the detection of a bony window for obvious reasons. Regarding CBCT imaging methods, specificity was higher for this particular defect due to the cross-sectional images provided for evaluation (12). Notably, the specificity differs among CBCT/N and CBCT/HR method in defect assessment. In general, the CBCT/HR imaging method showed the highest specificity in detecting PBDs. This can be only attributed to the different voxel size used (0.3 vs 0.125 for CBCT/N and CBCT/HR method respectively) due to the standardized conditions under which both CBCTs were obtained. This is coincident with the results of studies that have shown that decreasing the voxel size did not significantly improve sensitivity or specificity of PBDs detection (17,18,20,24,27).

Considering the ex-vivo nature of our study, we determined the sensitivity and specificity of three different imaging methods used in dental practice to evaluate peri-implant defects the nature of which is relatively common. For this reason, we based our results on observers of three different levels of expertise who showed that their opinion regarding the interpretation of defects does not differ significantly. Thus, we consider our choice to be pertinent in regard to accurately determine sensitivity and specificity of the methods evaluated.

An important limitation of our study is that data are not of clinical origin as in other studies such as the study by Serino *et al* (2023), in which two diagnostic digital intraoral radiographs were obtained, one prior to implant removal and the other six months following explantation (29). But the only way to avoid motion artifacts - that may reduce image quality - is only to perform in- or ex-vivo studies. Also, our data were acquired with a single FoV (12×8cm) chosen to match the size of the experimental setup, thus keeping scatter radiation at similar levels (30). At any rate, such studies using CBCT are susceptible in beam hardening artifacts (due to the implant presence) the severity of which is related to the voxel size and the exposure parameters used for each examination (18,20,27).

In conclusion, CBCT performs better than PA in induced PBDs. Our results, based on a homogenous sample of observers' expertise level, also showed excellent sensitivity and low specificity for PA imaging method. Thus, this can be considered as a very useful tool in the routine diagnostics in all clinical scenarios for the detection of peri-implant bone sites and specific bone defects. Although CBCT has excellent sensitivity and good specificity in imaging PBDs, this method should be used with caution for specific indications. Also taking into account the 2017's World Periodontology Workshop recommendations on the use of PAs after detection of clinical inflammation following peri-implant probing, we do not recommend the use of CBCT as standard imaging procedure for the evaluation of PBDs, mainly because of the increased radiation dose (7,11).

## Figures and Tables

**Table 1:** Distribution of characteristics of peri-implant bone defects submitted for evaluation, for each radiographic method separately, compared to the Gold Standard (GS).

Characteristics		GS	Imagine method used - Frequency (%)		
			CBCT/N n=48	CBCT/HR n=48	PA n=49
Bone defect	No	64 (44.1)	23 (47.9)	23 (47.9)	18 (36.7)
	Yes	81 (55.9)	25 (52.1)	25 (52.1)	31 (63.3)
Bony window on the buccal side of the crestal bone	Yes	16 (11.0)	4 (8.3)	4 (8.3)	8 (16.3)
	1mm, bony window	10 (62.5)	2 (50.0)	2 (50.0)	6 (75.0)
	2mm, bony window	6 (37.5)	2 (50.0)	2 (50.0)	2 (25.0)
	No	129 (89.0)	44 (91.7)	44 (91.7)	41 (83.7)
Bone recessions buccally to the implant side	Yes	20 (13.8)	6 (12.5)	6 (12.5)	8 (16.3)
	1mm, bone recession	8 (40.0)	2 (33.3)	2 (33.3)	4 (50.0)
	2mm, bone recession	12 (60.0)	4 (66.7)	4 (66.7)	4 (50.0)
	No	125 (86.2)	42 (87.5)	42 (87.5)	41 (83.7)
Intraosseous defects	Yes	20 (13.8)	7 (14.6)	7 (14.6)	6 (12.2)
	Four-wall	7 (35.0)	2 (28.6)	2 (28.6)	3 (50.0)
	Three-wall	13 (65.0)	5 (71.4)	5 (71.4)	3 (50.0)
	No	125 (86.2)	41 (85.4)	41 (85.4)	43 (87.8)
Circumferential defect	Yes	19 (76.0)	5 (62.5)	5 (62.5)	9 (100.0)
	Circumferential defect with buccal recession	6 (24.0)	3 (37.5)	3 (37.5)	0 (0.0)
	No	120 (82.8)	40 (83.3)	40 (83.3)	40 (83.3)
Inability to determine the presence or absence of a bone defect		0 (0)	0 (0)	0 (0)	0 (0)

GS: Gold Standard; CBCT/N: voxel size 0.3mm^3; ^CBCT/HR: voxel size0.125mm^3^; PA: Periapical radiograph.

**Table 2:** The overall detectability rate (for all imaging modalities used) of the peri-implant bone defects between observers and their level of expertise.

Characteristics		Gold Standard (GS)	Expertise - Frequency (%)								
			Oral Radiologists (OR)			Implantologists (IS)			General Practitioners (GP)		
			Observers								
			1	2	3	4	5	6	7	8	9
Bone defect	No	64 (44.1)	63 (43.5)	59 (40.7)	69 (47.6)	69 (47.6)	53 (36.6)	77 (53.1)	94 (64.8)	90 (62.1)	63 (43.4)
	Yes	81 (55.9)	82 (56.6)	86 (59.3)	76 (52.4)	76 (52.4)	92 (63.5)	68 (46.9)	51 (35.2)	55 (38.0)	84 (56.6)
Bony window on the buccal side of the crestal bone	Yes	16 (11.0)	5 (3.5)	4 (2.8)	5 (3.5)	10 (6.9)	5 (3.5)	5 (3.5)	8 (5.5)	4 (2.8)	5 (3.4)
	1mm, bony window	10 (62.5)	4 (80.0)	0 (0.0)	2 (40.0)	7 (70.0)	2 (40.0)	1 (20.0)	5 (62.5)	0 (0.0)	4 (80.0)
	2mm, bony window	6 (37.5)	1 (20.0)	4 (100.0)	3 (60.0)	3 (30.0)	3 (60.0)	4 (80.0)	3 (37.5)	4 (100)	1 (20.0)
	No	129 (89.0)	140 (96.5)	141 (97.2)	140 (96.6)	135 (93.1)	140 (96.5)	140 (96.5)	137 (94.5)	141 (97.2)	140 (96.6)
Bone recessions buccally to the implant side	Yes	20 (13.8)	12 (8.3)	20 (13.8)	12 (8.3)	19 (13.1)	6 (4.1)	22 (15.2)	48 (33.1)	49 (38.8)	12 (8.3)
	1mm, bone recession	8 (40.0)	8 (66.7)	6 (30.0)	8 (66.7)	13 (68.4)	1 (16.7)	18 (81.8)	32 (66.7)	25 (51.0)	8 (66.7)
	2mm, bone recession	12 (60.0)	4 (33.3)	14 (70.0)	4 (33.3)	6 (31.6)	5 (83.3)	4 (18.2)	16 (33.3)	24 (49.0)	4 (33.3)
	No	125 (86.2)	133 (91.7)	125 (86.2)	133 (91.7)	126 (86.9)	139 (95.9)	123 (84.8)	97 (66.9)	96 (66.2)	133 (91.7)
Intraosseous defects	Yes	20 (13.8)	19 (13.1)	11 (7.6)	24 (16.6)	14 (9.7)	18 (12.4)	26 (17.9)	11 (7.6)	13 (9.0)	19 (13.1)
	Four-wall	7 (35.0)	2 (10.5)	3 (27.3)	9 (37.5)	3 (21.4)	3 (16.7)	10 (39.5)	2 (18.2)	3 (23.1)	2 (10.5)
	Three-wall	13 (65.0)	17 (89.5)	8 (72.7)	15 (62.5)	11 (78.6)	15 (83.3)	16 (61.5)	9 (81.8)	10 (76.9)	17 (89.5)
	No	125 (86.2)	126 (86.9)	134 (92.4)	121 (83.5)	131 (90.3)	127 (87.6)	119 (82.1)	134 (92.4)	132 (91.0)	126 (87.9)
Circumferential defect	Yes	19 (76.0)	20 (74.1)	11 (61.1)	21 (75.0)	14 (70.0)	18 (78.3)	20 (74.1)	14 (66.7)	11 (45.8)	19 (61.3)
	Circumferential defect with buccal recession	6 (24.0)	7 (25.9)	7 (38.9)	7 (25.0)	6 (30.0)	5 (21.7)	7 (25.9)	7 (33.3)	13 (54.2)	12 (38.7)
	No	120 (82.8)	118 (81.4)	127 (87.6)	117 (80.7)	125 (86.2)	122 (84.4)	118 (81.4)	124 (85.5)	121 (83.5)	114 (78.6)
Inability to determine the presence or absence of a bone defect		0 (0)	0 (0.0)	6 (4.1)	0 (0.0)	6 (4.1)	1 (0.7)	0 (0.0)	6 (4.1)	0 (0.0)	4 (2.8)

**Table 3:** Results from comparing the rate of agreement: a) The Fleiss' Kappa/inter-examiner agreement (average %) assessment of the identification of the peri-implant defects between different observers with different expertise and the Gold Standard (GS), overall and within the same radiographic method, b) the p-values between observers of the same specialty, and between the imaging methods used (CBCT and PA), and c) the p-values between the specialties of the observers.

Expertise	Values	Observers	Overalln=145	CBCT/Nn=48	CBCT/HRN=48	PAn=49
OR	Kappa Agreement (%)	1	81.2	75.8	93.3	74.2
		2	82.2	85.1	88.7	72.7
		3	85.0	77.3	92.1	82.7
		All	82.8	79.4	91.4	76.5
	p-value ^1,2^	1 vs 2	-	0.961		0.208
		1 vs 3	-	0.589		0.084
		2 vs 3	-	0.623		0.791
IS	Kappa Agreement (%)	4	86.8	89.3	88.5	76.4
		5	71.1	72.0	74.7	66.2
		6	83.0	75.4	88.9	80.5
		All	80.3	78.9	84.0	74.4
	p-value ^1,2^	4 vs 5	-	0.140		0.106
		4 vs 6	-	0.859		0.443
		5 vs 6	-	0.192		0.831
GP	Kappa Agreement (%)	7	78.6	79.4	83.2	69.8
		8	71.4	71.4	79.7	64.4
		9	74.5	74.5	78.0	72.9
		All	74.8	75.1	80.3	69.0
	p-value ^1,2^	7 vs 8	-	0.540		0.190
		7 vs 9	-	0.910		0.232
		8 vs 9	-	0.616		0.702
OR vs IS	p-value ^3^	-	0.584	0.952	0.272	0.810
OR vs GP		-	0.096	0.617	0.121	0.407
IS vs GP		-	0.263	0.660	0.638	0.555

OR: Oral Radiologists; IS: Implantologists; GP: General Practitioners; CBCT/N: voxel size 0.3mm^3; ^CBCT/HR: voxel size 0.125mm^3^; PA: Periapical radiograph; 1: z-test for 2 proportions, between observers; 2: z-test for 2 proportions, between imaging methods; 3: z-test for 2 proportions.

**Table 4:** Sensitivity (%) and specificity (%) by imaging method.

Characteristics	CBCT/N (n=48)		CBCT/HR (n=48)		PA (n=49)	
	Sens. (%)	Spec. (%)	Sens. (%)	Spec. (%)	Sens. (%)	Spec. (%)
Bone defect	100.0	13.0	100.0	21.7	77.4	77.8
Bony window on the buccal side of the crestal bone	75.0	97.7	75.0	88.6	25.0	100.0
Bone recessions buccally to the implant side	100.0	35.7	100.0	50.0	100.0	34.2
Intraosseous defects	100.0	65.9	100.0	78.1	100.0	20.0
Circumferential defect	100.0	72.5	100.0	72.5	100.0	20.0
Total	95.0%	57.0%	95.0%	62.2%	80.5%	50.4%

CBCT/N: voxel size 0.3mm^3; ^CBCT/HR: voxel size 0.125mm^3^; PA: Periapical radiograph; Sens.: Sensitivity; Spec.: Specificity.
